# A swollen thumb

**DOI:** 10.1016/j.jdcr.2021.06.002

**Published:** 2021-06-10

**Authors:** Farah El Hadadi, Line Mezni, Kaoutar Znati, Mariame Meziane, Karima Senouci

**Affiliations:** a Department of Dermatology, Mohammed V University in Rabat, Ibn Sina University Hospital, Rabat, Morocco; b Department of Pathology, Mohammed V University in Rabat, Ibn Sina University Hospital, Rabat, Morocco

**Keywords:** finger pulp, giant cell tumor of tendon sheath, histiocytes, hemosiderin, CT, computed tomography, GCTTS, giant cell tumor of tendon sheath, MRI, Magnetic resonance imaging, PET, positron emission tomography

A 48-year-old, right-handed Moroccan woman with hepatitis C, receiving sofosbuvir 400 mg per day and daclatasvir 60 mg per day, presented 2 years ago a fixed painless flesh-colored nodule at the pulp of the right thumb.

There was no functional impairment or history of trauma, but the patient complained of discomfort during thumb movements due to the size of the nodule (Fig 1). The X-ray revealed a soft-tissue swelling without exostosis. Histologic examination of an excisional biopsy revealed variable proportions of mononuclear cells, osteoclast-like giant cells, foamy histiocytes, and hemosiderin-laden histiocytes (Figs 2 and 3).

**Fig 1:**
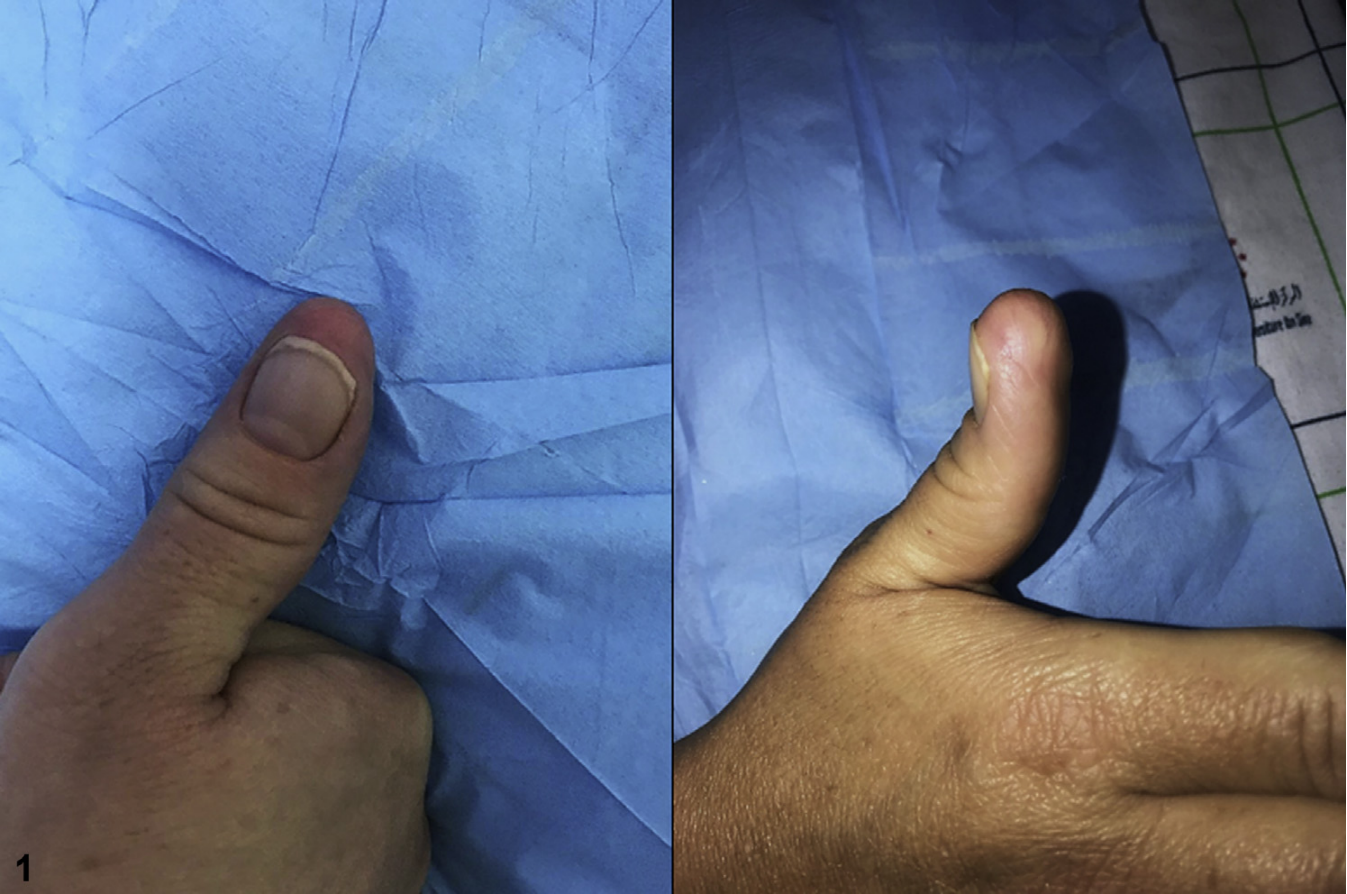

**Fig 2:**
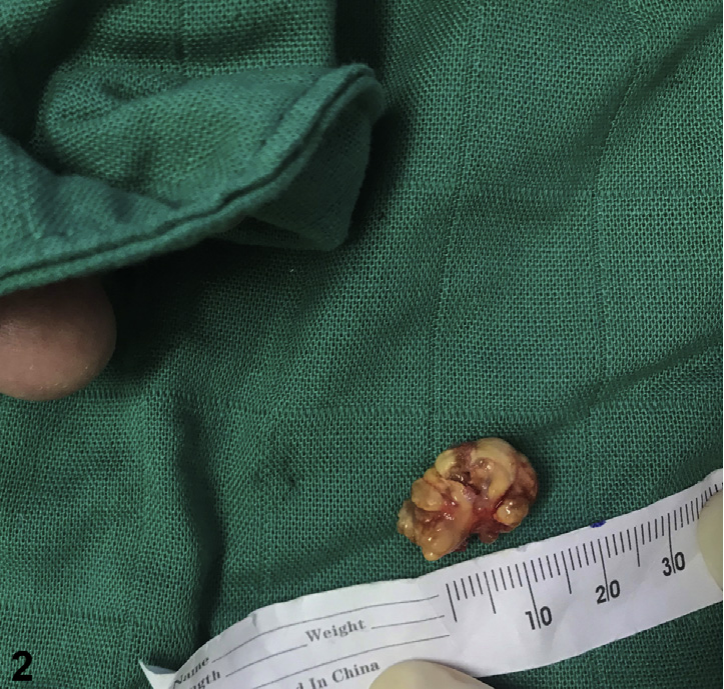

**Fig 3:**
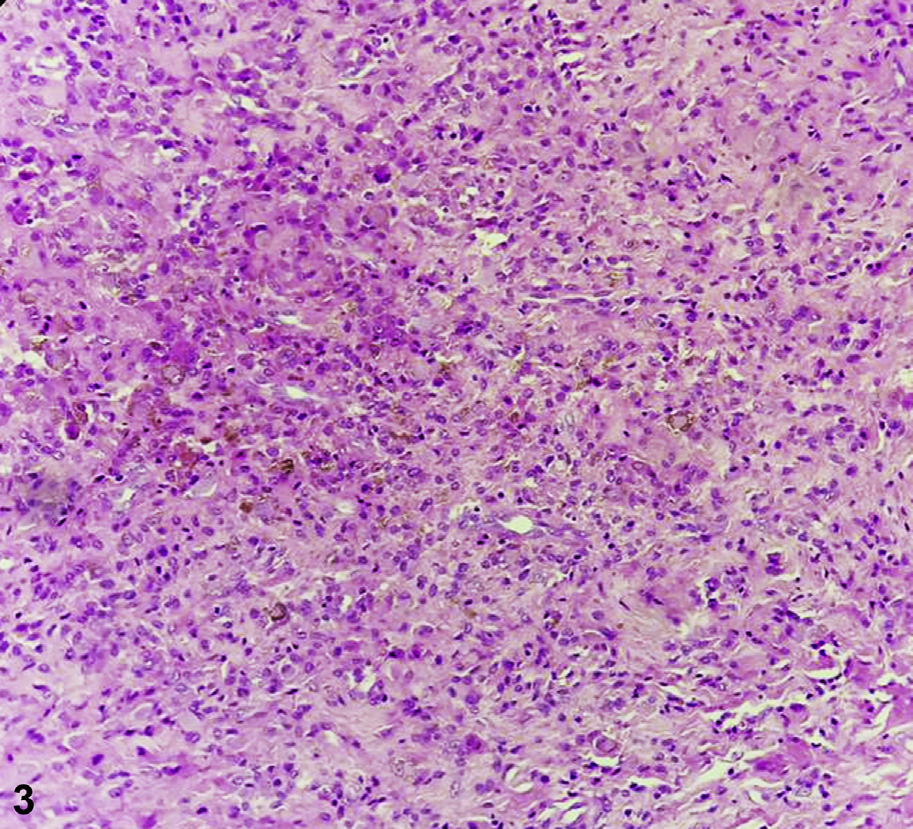

**Question 1: What is the most likely diagnosis?**

A. Lipoma
B. Rheumatoid nodule
C. Superficial acral fibromyxoma
D. Giant cell tumor of tendon sheath (GCTTS)
E. Tuberculous tenosynovitis

**Answers:**

A. Lipoma – Incorrect. Lipomas rarely present on the hand (<1%).^1^ They often arise in the thenar or hypothenar regions, and the histology shows lobule mature adipocytes traversed by fibrous tissue.
B. Rheumatoid nodule – Incorrect. First, the pulp of the thumb is not a location of rheumatoid nodules and the patient had no history of systemic disease or joint symptoms. These nodules are moveable in subcutaneous planes, which was not the case our patient, in whom the nodule was fixed. Finally, the histology of rheumatoid nodule exhibits a central area of necrosis surrounded by palisading epithelioid macrophages enclosed by granulation tissue containing lymphocytes and histiocytes.^2^
C. Superficial acral fibromyxoma – Incorrect. Superficial acral fibromyxoma is a rare, slow-growing myxoid tumor in the subungual area with a predilection for the great toe; nail involvement is seen in 50% of cases, usually with hyperkeratosis and onycholysis. Histologically, the tumor is composed of abundant myxoid stroma in the dermis with spindle-shaped cells.
D. GCTTS – Correct. GCTTS is the most common benign neoplasm of the hand.^3^ The tumor is firm, lobulated, nontender, slow-growing, and fixed to the underlying structures. Macroscopically, it appears as an encapsulated, polylobed, brownish-yellow tumor. Usually, it develops next to the proximal or distal interphalangeal and metacarpo-phlangeal joints.
E. Tuberculous tenosynovitis – Incorrect. Our patient was asymptomatic (no fever, painless nodule, no weight loss), and no caseous granulomas, tuberculosis bacillus, or multinucleated Langerhans giant cells were observed in the biopsy.

**Question 2: What is the most specific non-invasive test?**

A. Positron emission tomography (PET)
B. Ultrasound
C. Magnetic resonance imaging (MRI)
D. Computed tomography (CT)
E. Skin biopsy

**Answers:**

A. PET – Incorrect. PET with fluorine-18 fluorodeoxyglucose combined with computed tomography (PET/CT) is widely used to differentiate benign from malignant tumors and to detect distant metastasis. However, GCTTS may show high fluorine-18 fluorodeoxyglucose uptake, which can resemble the situation characteristic of a malignant tumor. Therefore, fluorine-18 fluorodeoxyglucose PET/CT has limited diagnostic value, due to its false-positive findings.
B. Ultrasound – Incorrect. Sonography is capable of distinguishing solid and cystic masses; however, GCTTS usually appears as a solid, homogeneous, hypoechoic mass. Internal vascularity can be demonstrated by Doppler examination in most cases, but does not have high specificity for GCTTS.
C. MRI – Incorrect. MRI typically shows a well-defined mass adjacent to or enveloping a tendon. Characteristically, the mass is hypointense on T1-weighted images. On T2-weighted images, there is usually low signal due to chronic hemorrhage with hemosiderin deposition. The most specific feature of GCTTS is the blooming artefact (hemosiderin deposit) on gradient echo sequences.^4^ However, MRI cannot distinguish the exact nature of the tumor (benign, malignant).
D. CT – Incorrect. Although it can reveal bone destruction and can be helpful in some cases of giant and infiltrative tumors, it is not the best diagnostic method for tendons and ligaments.
E. Skin biopsy – Correct. Histology confirms the diagnosis, revealing foci of hemosiderin-laden histiocytes with a predominance of small stromal mononuclear cells and numerous larger histiocytoid mononuclear cells with eccentric nuclei.

**Question 3: What is the best treatment option in this case?**

A. Abstention
B. Surgical excision
C. Radiotherapy
D. Pexidartinib
E. Surgery followed by radiotherapy

**Answers:**

A. Abstention – Incorrect. Although there was no functional impairment, the tumor was increasing in size, causing discomfort. Moreover, some diffuse types of GCTTS tend to be more aggressive and are capable of malignant transformation.
B. Surgical excision – Correct. Complete local excision is the treatment of choice. The main concern regarding this treatment is related to the high recurrence rates. Besides incomplete excision, there is no consensus concerning the effect of other risk factors on recurrence.
C. Radiotherapy – Incorrect. The use of radiotherapy is limited, it is purported to be useful for controlling infiltrative cases and preventing recurrence.
D. Pexidartinib – Incorrect. Pexidartinib was approved by the Food and Drug Administration in 2019 for adult patients with symptomatic tenosynovial giant cell tumor associated with severe morbidity or functional limitations and for surgically unresectable pigmented villonodular synovitis (large variant of GCTTS),^5^ none of which were present in our case (small unique nodule); moreover, pexidartinib can cause liver toxicity, and since our patient was receiving treatment for hepatitis C, this treatment was not recommended for her.
E. Surgery and radiotherapy – Incorrect. There is no indication for radiotherapy after surgery in this case. In fact, it is the first episode in our patient and the tumor was encapsulated and disconnected from the tendon.

## Conflicts of interest

None declared.
